# B-Type Natriuretic Peptides and High-Sensitive Troponin I as COVID-19 Survival Factors: Which One Is the Best Performer?

**DOI:** 10.3390/jcm10122726

**Published:** 2021-06-21

**Authors:** Renato de Falco, Maria Vargas, Daniela Palma, Marcella Savoia, Anna Miscioscia, Biagio Pinchera, Martina Vano, Giuseppe Servillo, Ivan Gentile, Giuliana Fortunato

**Affiliations:** 1 Department of Biochemistry and Medical Biotechnology, University of Naples “Federico II”, Via Sergio Pansini 5, 80131 Naples, Italy; renato.defalco@unina.it (R.d.F.); palmad@ceinge.unina.it (D.P.); marcella.savoia@unina.it (M.S.); anna_miscioscia@yahoo.it (A.M.); martina.vano@unina.it (M.V.); 2 Department of Neuroscience, Reproductive and Odontostomatological Sciences, University of Naples “Federico II”, Via Sergio Pansini 5, 80131 Naples, Italy; maria.vargas@unina.it (M.V.); giuseppe.servillo@unina.it (G.S.); 3 CEINGE Biotecnologie Avanzate s.c. a r.l., Via Gaetano Salvatore 486, 80145 Naples, Italy; 4 Department of Clinical Medicine and Surgery, Section of Infectious Diseases, University of Naples “Federico II”, Via Sergio Pansini 5, 80131 Naples, Italy; biapin89@virgilio.it (B.P.); ivan.gentile@unina.it (I.G.); 5 Staff UNESCO Chair for Health Education and Sustainable Development, University of Naples Federico II, Via Sergio Pansini 5, 80131 Naples, Italy

**Keywords:** COVID-19, BNP, NT-proBNP, troponin, prognostic markers, fatal outcome

## Abstract

Increased concentrations of B-type natriuretic peptide (BNP), N-terminal pro-B-type natriuretic peptide (NT-proBNP) and high-sensitivity troponin I (HsTnI) in COVID-19 patients have already been reported. The aim of this study is to evaluate which of these common markers of cardiac disease is the most useful predictor of fatal outcome in COVID-19 patients. One hundred and seventy-four patients affected with COVID-19 were recruited, and markers of cardiac disease and the clinical history of the patients were collected at admission in the infectious disease unit or intensive care unit. NT-proBNP, BNP and HsTnI values were higher in in-hospital non-surviving patients. Receiver operating characteristic (ROC) curve analysis of NT-proBNP, BNP and HsTnI was performed, with NT-proBNP (AUC = 0.951) and HsTnI (AUC = 0.947) being better performers (*p* = 0.01) than BNP (AUC = 0.777). Logistic regression was performed assessing the relation of HsTnI and NT-proBNP to fatal outcome adjusting for age and gender, with only NT-proBNP being significant. The population was then divided into two groups, one with higher NT-proBNP values at admission than the cut-off resulted from the ROC curve (511 ng/L) and a second one with lower values. The Kaplan–Meier analysis showed an absence of fatal outcome in the group of patients with NT-proBNP values lower than the cut-off (*p* < 0.001). NT-proBNP proved to be the best prognostic tool for fatal outcome among markers of cardiac disease in COVID-19 patients.

## 1. Introduction

In the last days of December 2019, coronavirus disease 2019 (COVID-19), which is caused by severe acute respiratory syndrome coronavirus 2 (SARS-CoV-2), broke out in the Huabei region (China). Since then, SARS-CoV-2 has continued to spread worldwide leading the WHO, on March 11th, to declare COVID-19 a pandemic [1]. Patients affected by COVID-19 gave rise to high rates of hospitalization and intensive care unit (ICU) admissions [2], with in-hospital mortality as high as 28% in severe COVID-19 [3].

Several studies investigated the possible correlation between markers of cardiac disease and the worsening of COVID-19 severity [4,5]. Diffuse inflammation is the hallmark of the disease and could possibly lead to atherosclerosis-based cardiovascular disease, as previously demonstrated for other infectious diseases [6]. Increased concentrations of markers for cardiac disease were detected early in patients suffering from COVID-19, and in particular high concentrations of high-sensitive cardiac troponin I (HsTnI) and N-terminal pro-B-type natriuretic peptide (NT-proBNP) were associated with the worst prognosis for hospitalized patients [7,8,9]. NT-proBNP is currently known as an independent risk factor for in-hospital death of patients with severe COVID-19 [10]. B-type natriuretic peptide (BNP) was recently reported as a possible predictive marker for survival of COVID-19 patients [11]. HsTnI is significantly increased at admission in patients with severe COVID-19 compared to those affected by milder forms of COVID-19 [12], and has been recently described as a possible predictor of in-hospital mortality [13].

The aim of this study was to evaluate which of BNP, NT-proBNP and HsTnI could be considered the best prognostic predictor in COVID-19 patients.

## 2. Materials and Methods

This prospective observational study was performed according to the current version of the Helsinki Declaration and was approved by the Ethical Committee of the University of Naples “Federico II”, Italy (Number 156/20, 22 April 2020). Informed consent was obtained from all subjects involved in the study.

Patients were hospitalized in two units of the University of Naples “Federico II” (infectious disease unit and ICU) directly from the emergency room or following general practitioner indications. In order to be selected, all patients admitted to those wards from 10 March 2020 to 30 April 2020, and from 8 December 2020 to 25 January 2021 had to match the following inclusion criteria: intention to participate in the study and signature of informed consent, age > 18 years, and rhino-oropharyngeal swab positivity for SARS-CoV-2 RNA. Exclusion criteria included inability to understand or sign informed consent, age < 18 years, absence of positivity for SARS-CoV-2 RNA test, positivity for serological tests only, as well as any other condition that in the investigator’s opinion could make the patient non-eligible to participate in the study or could interfere with the patient’s participation in the study and its completion. 

BNP, NT-proBNP and HsTnI were analyzed at admission. Data on the clinical course of COVID-19 before hospitalization and its related complications were also acquired. In particular, the presence of pre-existing diseases related to cardiac and pulmonary functions was recorded and included in data analysis, alongside with diabetes and hypertension.

BNP and HsTnI plasma concentrations were measured by chemiluminescent microparticle immunoassay on the ARCHITECT i2000SR system (Abbott Laboratories, Wiesbaden, Germany). NT-proBNP was also measured on the ARCHITECT i2000SR system by using the Alere assay (Roche Diagnostics GmbH, Mannheim, Germany). BNP and NT-proBNP were collected in tubes with EDTA, while HsTnI was collected in tubes with sodium heparin. 

Upper reference ranges for BNP were: 89 ng/L for patients under 45 years, 111 ng/L for 45–54 years-old patients, 155 ng/L for 54–64 years-old patients, 159 ng/L for 64–74 years-old patients and 266 ng/L for patients > 74 years. Upper reference ranges for NT-proBNP were 125 ng/L for patients < 75 years and 450 ng/L for patients > 75 years. The decision level (99th percentile) for HsTnI was 34 ng/L for men and 16 ng/L for women.

Data analysis was performed comparing the level of all the markers between in-hospital survivors who were successfully discharged and in-hospital non-survivors, for both critical patients (ICU) and non-critical patients (infectious disease unit).

The normal distribution of variables was evaluated by the Kolmogorov–Smirnov test and the chi-square test to compare frequencies between groups, with the use of the Fisher exact test when comparing dichotomic variables. Categorical variables are reported as number of subjects and percentages, and continuous non-parametric variables are reported as interquartile range, respectively. Differences between two groups were assessed by the Mann–Whitney test.

Analysis of receiver operating characteristic (ROC) curves and dot diagrams was performed with the MedCalc software Version 11.5.1 (Medcalc Software Ltd., 8400 Ostend, Belgium). Statistical significance of the area under the ROC curves (AUC) was calculated against the null hypothesis AUC = 0.5 as recommended by DeLong et al. [14]. Threshold values were determined by the farthest point from the bisector of the ROC curve.

Binary logistic regression was performed, including gender (encoded as 1 = male and 2 = female), age in years, NT-proBNP and HsTnI. Kaplan–Meier analysis (log-rank test) was used to compare the survival experience of the participants. Statistical analyses were carried out using the statistical Predictive Analytics Software version 21.0 (SPSS Inc., Chicago, IL, USA). A *p*-value of *p*  < 0.05 was considered statistically significant.

## 3. Results

One hundred and seventy-four patients were enrolled in this study: eighteen critical patients were admitted to the ICU while 156 non-critical patients were hospitalized in the infectious disease unit. Thirteen patients showed in-hospital fatal outcome, ten from the ICU and three from the infectious disease unit. The direct causes of death were respiratory failure (*n* = 3), septic complications (*n* = 4), neurological complications (*n* = 2) and cardiogenic shock (*n* = 4). Population characteristics, biochemical baseline values and differences between surviving and non-surviving groups are depicted in Table 1.

Comorbidities, smoking and oxygen saturation at admission were not statistically different between surviving and non-surviving patients. In detail, cardiac pathologies reported were ischemic heart disease (*n* = 14), heart failure (*n* = 6) and atrial fibrillation (*n* = 4). Older age, a higher percentage of male gender, and higher concentrations of BNP, NT-proBNP and HsTnI were present in the non-surviving group. Only 17 patients showed baseline values higher than the appropriate upper reference ranges for age and sex for BNP (9,8%) and 20 for HsTnI (11.5%), whereas higher baseline values for NT-proBNP were reported in 87 patients (50.0%). 

**Figure 1:**
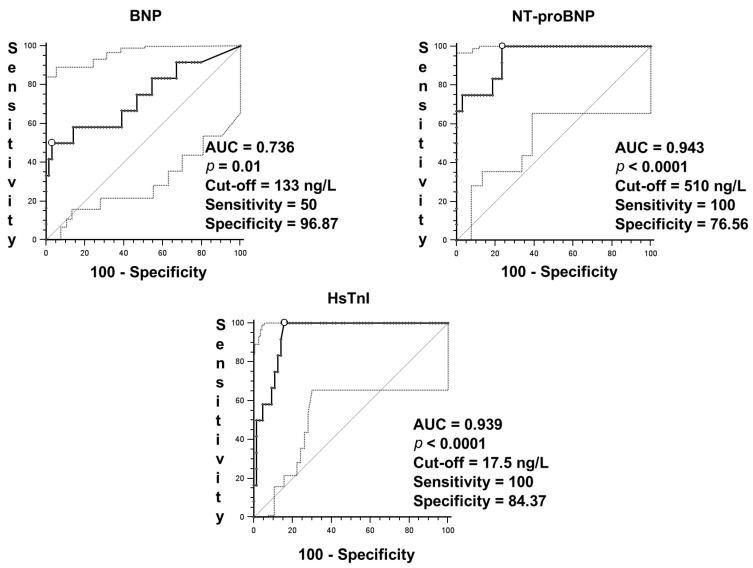
ROC curves for NT-proBNP, BNP and HsTnI concentrations at admission. The receiver operating characteristic (ROC) curve is indicated with a line in bold and full circles represent the criterion points. The open circle indicates the cut-off point. The light line indicates the bisector and dotted lines indicate 95% confidence intervals.

ROC curves were performed (Figure 1) for BNP (AUC = 0.777, *p* < 0.001), NT-proBNP (AUC = 0.951, *p* < 0.001) and HsTnI (AUC = 0.974, *p* < 0.001), with both NT-proBNP and HsTnI ROC curves performing better than the BNP ROC curve (*p* = 0.01). There were no statistical differences between NT-proBNP and HsTnI ROC curves.

Logistic regression was then performed with age, gender, NT-proBNP and HsTnI as covariates and in-hospital death as the dependent variable, with only NT-proBNP showing a significant odds ratio (OR = 1.002, 95%CI 1.001–1.003, *p* = 0.04). 

**Figure 2:**
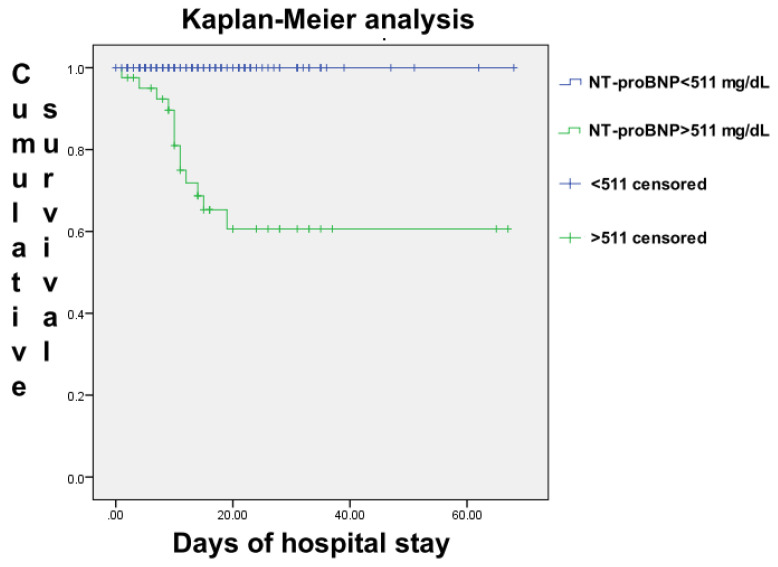
Kaplan–Meier survival analysis between groups with highest value of NT-proBNP ratio higher or lower than 95 during hospital stay. Statistical difference evaluated with log-rank test.

The population was then divided into two groups based on whether patients had higher values than the cut-off point of the NT-proBNP ROC curve (511 ng/L) or not. The Kaplan–Meier analysis was performed (Figure 2) and logarithmic rank analysis showed a statistical difference in terms of survival between the two groups (*p* < 0.001), with an absence of fatal outcome in the group of patients with NT-proBNP values lower than 511 ng/L at admission.

## 4. Discussion

This prospective observational study highlighted that NT-proBNP may be the most useful predictor of fatal outcome in COVID-19 patients. There was no fatal outcome in patients with a value of NT-proBNP below the 511 ng/L cut-off resulting from the ROC curve at admission. Moreover, a large number of patients in both surviving and non-surviving groups showed pathological values of NT-proBNP above the upper reference interval, although this reference interval was age adjusted [15,16]. HsTnI, nonetheless, seems to be a good mortality predictor as well.

BNP and NT-proBNP are secreted in response to increased myocardial wall stress [17]. Quite surprisingly, despite BNP values being higher in non-surviving patients, the NT-proBNP ROC curve showed that NT-proBNP was a more effective prognostic tool than BNP, although these two markers may be considered as equal diagnostic tools for heart failure [18]. This discordance may be due to extrarenal clearance in the context of renal failure, since neutral endopeptidases seem to accumulate, whereas up-regulation of the type-C natriuretic peptide receptor seems to occur in critical states [19]. Another explanation may be related to the presence of NT-proBNP glycosylation, a process which may be inhibited during critical status; NT-proBNP is often miscalculated due to its preponderant undetectable glycosylated quota [20], showing much higher values when tubes are pre-treated with de-glycosylation enzymes [21]. Further studies are needed to support this hypothesis. Finally, this result may be caused by a proBNP cross-reaction occurring during NT-proBNP detection without affecting the BNP measurement [22], leading to NT-proBNP values higher than BNP values.

BNP was recently described in the meta-analysis by Wungu et al. [11] as a possible predictive marker for survival of COVID-19 patients. A significant difference between surviving and non-surviving patients was reported in our study as well, but BNP was eventually outperformed by NT-proBNP and HsTnI ROC curves.

Elevated NT-proBNP concentrations were associated to a significant risk of mortality in septic patients even without a clear cardiac depression [23]. Several mechanisms are likely to account for increased NT-proBNP in septic patients, including acute renal injury and proinflammatory molecules such as lipopolysaccharides, interleukin 1, C-reactive protein, and cardiotrophin I, which are independent of ventricular function [24,25]. 

The angiotensin-converting enzyme 2 (ACE2) is likely to play a role, being a major binding site for severe acute respiratory syndrome coronavirus 2 (SARS-CoV-2) [26]. As an indirect mechanism, the down-regulation of ACE2 and the subsequent activation of the renin–angiotensin–aldosterone system induced by the viral infection may contribute to the local and systemic inflammatory response, which damages the tissue and triggers the release of biomarkers [26]. As a direct mechanism, SARS-CoV-2, binding ACE2, prevents the angiotensin II clearance, by reducing the concentrations of angiotensin-(1–7); thus, resulting in an increase in NT-proBNP concentrations and kidney podocytes dysfunction [26,27].

HsTnI concentrations were higher in non-surviving patients, and its ROC curve was comparable to the NT-proBNP one. When age and sex were considered, though, the still significant variable was only NT-proBNP. HsTnI, in fact, is highly influenced by gender [28], thus, making NT-proBNP a highly, very reliable independent prognostic factor at admission for fatal outcome in COVID-19 patients.

This is the first study to effectively compare BNP and NT-proBNP, which—as previously stated—are known as equal diagnostic tools for heart failure.

The study has a few limitations: only 18 patients were hospitalized in the ICU, whereas 156 patients were hospitalized in the infectious disease unit, leading to a heterogeneity in our population. Moreover, only six ICU patients showed in-hospital survival, as most of them were non-surviving patients. A greater number of patients discharged from the ICU might have been useful in order to compare these two different population subgroups.

If confirmed by larger population studies, our findings may provide clinicians with the most appropriate tools to gain relevant insight into COVID-19 prognosis and survival rate.

## 5. Conclusions

In conclusion, NT-proBNP turned out to be a better prognostic marker than BNP and HsTnI in COVID-19 disease. In particular, our study highlighted that a value of NT-proBNP below the cut-off of 511 ng/L at admission led to no in-hospital mortality in our population.

## Figures and Tables

**Table 1:** Population description: values at admission and difference between non-surviving and surviving patients.

	Total (*n* = 174)	Non-Surviving (*n* = 13)	Surviving (*n* = 161)	Significance
Age, years	55 (34–69)	79 (69–85)	50 (33–66)	*p* < 0.001
Smoking, %	40 (22.4)	1 (7.7)	39 (24.2)	ns
O_2_ Saturation, %	93 (90–96)	90 (87,5–95)	93 (90–96)	ns
No. of males, %	84 (48.3)	10 (76.9)	74 (46.0)	*p* = 0.043
Diabetes, %	28 (19.2)	2 (18.2)	26 (19.3)	ns
Cardiac pathologies, %	24 (13.7)	4 (36.4)	20 (12.4)	ns
COPD, %	16 (11.0)	2 (18.2)	14 (10.4)	ns
Hypertension, %	34 (23.1)	4 (36.4)	30 (22.1)	ns
BNP, ng/L	28.55 (12.38–78.45)	140.00 (24.38–310,98)	26.75 (11.83–58.95)	*p* < 0.001
NT-proBNP, ng/L	243.1 (77.78–810.88)	5599.60 (1022.13–11,503.30)	176.15 (59.40–505.60)	*p* < 0.001
HsTnI, ng/L	4.8 (2.2–24.3)	96.5 (34.0–261.0)	4.00 (1.8–13.3)	*p* < 0.001

Abbreviations: O_2_, oxygen; COPD, chronic obstructive pulmonary disease; BNP, brain natriuretic peptide; NT-proBNP, N-terminal pro-B-type natriuretic peptide; HsTnI, high-sensitive cardiac troponin I; ns, non-significant. Differences between two groups were assessed using Mann–Whitney test or Fisher’s exact test as appropriate. Continuous non-parametric variables are reported as median and interquartile range. Categorical variables are reported as number (percentage).

## Data Availability

The data presented in this study are available on request from the corresponding author.
